# Proton Pump Inhibitors and Dementia: Physiopathological Mechanisms and Clinical Consequences

**DOI:** 10.1155/2018/5257285

**Published:** 2018-03-21

**Authors:** Gloria Ortiz-Guerrero, Diana Amador-Muñoz, Carlos Alberto Calderón-Ospina, Daniel López-Fuentes, Mauricio Orlando Nava Mesa

**Affiliations:** ^1^Individualized Research Learner Program, Neuromuscular Research Division, University of Kansas Medical Center, 3901 Rainbow Blvd., Kansas City, KS 66160, USA; ^2^Neuroscience (NEUROS) Research Group, School of Medicine and Health Sciences, Universidad del Rosario, Carrera 24 No. 63C–69, Bogotá 111221, Colombia; ^3^Unidad de Farmacología, School of Medicine and Health Sciences, Universidad del Rosario, Carrera 24 No. 63C–69, Bogotá 111221, Colombia; ^4^Medical Social Service, Hospital de San Francisco, Kra 8 No. 6A–121, Gacheta 251230, Colombia

## Abstract

Alzheimer's disease (AD) is the most common type of dementia, mainly encompassing cognitive decline in subjects aged ≥65 years. Further, AD is characterized by selective synaptic and neuronal degeneration, vascular dysfunction, and two histopathological features: extracellular amyloid plaques composed of amyloid beta peptide (A*β*) and neurofibrillary tangles formed by hyperphosphorylated tau protein. Dementia and AD are chronic neurodegenerative conditions with a complex physiopathology involving both genetic and environmental factors. Recent clinical studies have shown that proton pump inhibitors (PPIs) are associated with risk of dementia, including AD. However, a recent case-control study reported decreased risk of dementia. PPIs are a widely indicated class of drugs for gastric acid-related disorders, although most older adult users are not treated for the correct indication. Although neurological side effects secondary to PPIs are rare, several preclinical reports indicate that PPIs might increase A*β* levels, interact with tau protein, and affect the neuronal microenvironment through several mechanisms. Considering the controversy between PPI use and dementia risk, as well as both cognitive and neuroprotective effects, the aim of this review is to examine the relationship between PPI use and brain effects from a neurobiological and clinical perspective.

## 1. Introduction

Dementia is a clinical syndrome that represents a wide spectrum of cognitive dysfunction and leads to progressive and chronic deterioration of social and occupational activities. According to the World Alzheimer Report, over 46.8 million people worldwide lived with dementia in 2015, with a predicted increase of cases to 74.7 million by 2030 and 131.5 million by 2050. In addition, 63% and 68% of all people with dementia will live in low- and middle-income countries by 2030 and 2050, respectively [1]. Because of this increased number of cases, the high cost of dementia is another issue that health systems will be dealing with in the future. Currently, the cost is estimated at $18 billion per year in the US, with an increase expected over upcoming years. Owing to the economic and social impact caused by dementia, the World Health Organization designated dementia a public health priority [2]. There are different types of dementia, with Alzheimer's disease (AD) being the most prevalent in humans, accounting for 50–70% of all cases [3]. The prevalence rate for AD increases predominantly with age, surging from 3.5% in people aged 75 years old to 46.3% in people aged 95 years old or older [4]. The histopathological hallmarks of AD include extracellular deposition of amyloid-*β* (A*β*) plaques, formation of neurofibrillary tangles (NFTs) from hyperphosphorylated tau protein, and neurodegeneration caused by progressive loss of neurons and their processes [5]. Moreover, the most accepted theory to explain the pathogenesis of AD is the amyloid hypothesis, which states that the cognitive disease phenotype is due to A*β* dyshomeostasis [6].

Proton pump inhibitors (PPIs) are a class of drugs used to treat gastric acid-related disorders, such as gastroesophageal reflux and peptic ulcer disease, and which act mainly as irreversible inhibitors of the H^+^/K^+^-ATPase pump to decrease gastric acid production [7]. PPIs have an excellent safety profile and have become one of the most prescribed drugs in recent years. According to the National Health and Nutrition Examination Survey, from 1999 to 2012, the percentage of adults aged 40–60 who received a prescription for PPIs almost doubled from 4.9% to 8.3% in the United States, surging concerns about their widespread use among this age group [8, 9]. Furthermore, various studies have shown that 50–70% of patients prescribed PPIs do not have the correct indication, especially in hospitalized elderly patients [10–12]. Overall, long-term use of PPIs has increased, leading to potential adverse effects such as nutritional deficiencies (vitamin B_12_, magnesium, and iron), renal damage, osteoporotic fracture, infection by *Clostridium difficile*, rhabdomyolysis, anemia, and thrombocytopenia [13]. Because of these adverse effects, their safety and role in cognitive function (including risk of developing dementia and AD) have been questioned lately. Several studies described association between PPIs and greater risk of developing dementia and AD in older people [9, 14, 15]. However, other study has not shown that PPIs were associated with greater risk of dementia neither AD [7]. In addition, a recent case-control study conducted in German primary care patients reported decreased risk of dementia with PPI use [16]. In fact, neuroprotective effects of PPIs have been recently described [17, 18]. Due to these controversial findings, and also the role of PPIs in progression from mild to severe cognitive dysfunction, the aim of this article is to review the relationship between PPI use and basic mechanisms of neuronal dysfunction. In this regard, we discuss if PPI use is associated with greater susceptibility to developing dementia, focusing on a neurobiological basis of AD. Consequently, we propose new hypothesis regarding the physiopathological mechanisms of cognitive impairment induced by acute and chronic PPI use and examine some associated factors that increase dementia susceptibility after PPI exposure.

## 2. The Effect of PPIs on the Central Nervous System

One of the human genes encoding H^+^/K^+^-ATPase (ATP12A/ATP1AL1) is expressed in the brain, colon, and placenta, while the other gene (ATP4A) is only expressed in gastric epithelial cells [19]. Accordingly, there is evidence of H^+^/K^+^-ATPase activity in the central nervous system (CNS), with certain isoforms expressed [20]. Proton pumps have several physiological functions in neurons and contribute to acid–base and potassium homeostasis [19]. Vesicular proton pumps (H^+^-ATPases or V-ATPases) create the proton gradient that is required for packaging of neurotransmitters into synaptic vesicles. Furthermore, new evidence indicates that vesicular H^+^/K^+^-ATPase plays an interesting role in both exocytosis and endocytosis in nerve terminals [21, 22]. Ca^2+^-ATPase, Na^+^/K^+^-ATPase, and H^+^/K^+^-ATPase are included in the PII subfamily of P-type ATPases [23]. P-type ATPases share common structural motifs and likely arise from a common ancestral gene [19]. Interestingly, the primary structure of the *α* subunit of gastric H^+^/K^+^-ATPase is 98% homologous within species and highly homologous to the catalytic subunit of Na^+^/K ^+^-ATPase (~63%) and sarcoplasmic/endoplasmic reticulum Ca^2+^-ATPase (SERCA) (~25%) [24, 25].

Proton pump inhibitors (e.g., omeprazole, lansoprazole, dexlansoprazole, rabeprazole, pantoprazole, and esomeprazole) effectively block acid secretion by covalent and irreversible binding to H^+^/K^+^-ATPases on the luminal surface of the parietal cell membrane [26, 27]. The site of reaction on the enzyme differs according to the particular PPI. However, all PPIs react with cysteine 813 in the active E2 configuration (ion-site-out) [27]. Considering the high homology between P-type ATPases, it is possible that PPIs can inhibit other ionic pumps in different organs or even induce systemic physiological changes. Indeed, the CNS may be one system affected, with its interaction facilitated by pathological conditions exhibiting reduced pH in the brain, cerebrospinal fluid, and blood (i.e., metabolic stress).

Passage of PPIs through the blood-brain barrier (BBB) has been calculated. After administering 10 mg/kg intravenous (IV) omeprazole to male Sprague Dawley rats, the area under the curve (AUC) of concentration versus time in the brain divided by AUC in blood was calculated [28]. The resulting blood-to-brain distribution coefficient was 0.15, indicating that up to 15% of a single IV dose of omeprazole can reach the CNS and potentially affect cognitive function with either acute or repetitive long-term use. Corroboratively, *in vitro* and *in vivo* pharmacokinetic studies have shown that lansoprazole may also penetrate the BBB [29].

Some PPIs, such as lansoprazole, esomeprazole, and pantoprazole, are reported to cause adverse neurological effects, mainly headaches [30, 31] and dizziness/vertigo [32]. Other adverse effects that involve the CNS (at a frequency of <1%) include depression, diplopia, disturbed sleep, drowsiness, insomnia, nervousness, and tremor. There have also been reports of sensoperceptual abnormalities (i.e., hallucinations) [33, 34] and delirium [35]. Neurological side effects induced by chronic PPI use may be related to indirect systemic abnormalities (i.e., magnesium and vitamin B_12_ deficiency) [36] or direct effects on neurons after passage through the BBB. Although the exact mechanisms on brain circuits have not been fully described, most neurological side effects are reported with chronic administration of PPIs.

## 3. PPIs and Physiopathological Effects in Dementia

PPI drugs can facilitate tau and A*β*-induced neurotoxicity, which may increase AD progression and cognitive decline. Below, we discuss relevant physiopathological mechanisms.

### 3.1. PPIs and A*β* Plaques

One of the most described effects in dementia relates to increased production of A*β* plaques by PPIs [37]. As already stated, one of the major hallmarks of AD is extracellular accumulation of A*β* plaques, which lead to oxidative and inflammatory damage in the brain [3]. These A*β* species are produced by cleavage of amyloid precursor protein (APP) by *β*-secretase (also known as *β*-site APP-cleaving enzyme 1 [BACE1]) and *γ*-secretase [38]. Although the total number of A*β* plaques does not correlate well with AD severity, there is a direct effect on cognition and cell death in APP/tau transgenic mice because of neuronal loss and the astrocyte inflammatory response [39]. In 2013, Badiola et al. [37] investigated the effect of PPIs on A*β* production using cell and animal models and suggested a novel hypothesis: they suggested that PPIs act as inverse *γ*-secretase modulators (iGSM), which change the *γ*-secretase cleavage site and thereby increase A*β*42 levels and decrease A*β*38 levels. In addition, PPIs can increase BACE1 activity, raising production of A*β*37 and A*β*40 levels. In AD, the major pathological species is thought to be A*β*42, but the most produced is A*β*40 [38]. Ultimately, PPIs (specifically lansoprazole) may alter media pH, amplifying the activity of other proteases, such as memprin-*β*, and generating A*β*2-x peptides (e.g., A*β*2-37, A*β*2-40, and A*β*2-42 species). Moreover, Badiola et al. were able to demonstrate that lansoprazole enhances A*β* production using *in vivo* and *in vitro* models, supporting the theory that PPIs effect AD by boosting A*β* production [37]. It has also been shown that PPIs can inhibit vacuolar proton pumps, which acidify lysosomes by pumping protons from the cytoplasm to the lumen of vacuoles in microglia and macrophages [40, 41]. Normally, this acid environment in lysosomes permits degradation of fibrillary A*β*. As PPIs can cross the BBB, they act on V-ATPases in an inhibitory manner, causing less degradation of fibrillary A*β* and hence a reduction in its clearance [28, 41]. To date, there are few studies that explain the relationship between the effects of PPIs and presence of A*β* plaques. It would be interesting if future studies determine why A*β* plaque production increases or their clearance decreases with PPI use.

### 3.2. PPIs and Tau Protein

Currently, AD diagnosis is based on neuropsychological tests (cognitive criteria), neuroimaging (i.e., MRI and amyloid deposits by PET), and tau/amyloid in CSF (biomarker criteria) that rule out other causes of dementia [42–44]. However, a definitive diagnosis can only be confirmed histopathologically by the extensive presence of A*β* and NFTs in the neocortex of postmortem brain tissue [45]. The main component of NFTs is paired helical filaments (PHFs) formed from hyperphosphorylated tau protein [46, 47]. Tau protein plays an important role as a microtubule-associated protein in neuronal axons, stabilizing microtubules and inducing their assembly [48]. When tau protein is hyperphosphorylated, it is unable to bind and stabilize microtubules, which leads to degeneration of affected neurons [49]. According to the neuroimmunomodulation theory of AD, the earliest CNS changes before the clinical onset of AD result from a chronic inflammatory response, which leads to abnormal tau phosphorylation and induces formation of PHFs and tau protein aggregates, ultimately resulting in cytoskeletal alterations [50]. Consequently, these lesions are present before the presentation of clinical symptoms of AD [51]. The first NFT lesion appears in the transentorhinal cortex and is proceeded by the entorhinal cortex and hippocampus and finally the neocortex [52]. Several studies have shown that NFTs correlate with cognitive decline and severity in AD, positioning tau NFTs as suitable targets for therapy and diagnosis in AD patients. Several researchers have focused on developing different radiotracer components based on their high affinity for tau protein. This will allow future quantification of tau burden using noninvasive diagnostic imaging such as positron emission tomography (PET) [53]. Okamura and coworkers have found that PPIs show high affinity to tau protein. They screened more than 2000 compounds to develop agents for use in PET and identified quinoline and benzimidazole as high affinity components of NFTs rather than senile plaques [51]. Subsequently, Rojo and colleagues found that lansoprazole, a Food and Drug Administration- (FDA-) approved PPI with a benzimidazole ring structure, had nanomolar binding affinity for tau aggregates, in agreement with Okamura's findings. They also showed that lansoprazole has high lipophilicity and can cross the BBB, reaching the brain within 37 min after administration and therefore showing suitability as a radiotracer for PET imaging. However, during kinetic analysis, lansoprazole interactions with tau NFTs did not fit the classical one-site or two-site binding models [29]. We hypothesize that this can be explained by the six tau protein isoforms expressed in the human CNS. These tau isoforms are divided into two sets with three (3R) and four (4R) microtubule-binding domains. In usual conditions, both sets of isoforms are expressed in an equal ratio, while under pathological conditions, different tauopathies show different isoform ratios with diverse morphologies [54]. Additionally, tau undergoes multiple posttranslational changes resulting in conformational modifications in aggregates, as well as alterations in binding affinities and binding sites of tau protein [55]. Further, Rojo et al. performed docking studies and identified strong hydrogen bond interactions between the NH group of the benzimidazole ring of lansoprazole and the C-terminal hexapeptide (386TDHGAE391) of the tau core [29]. Corroboratively, studies by Fawaz and coworkers determined that replacing the NH group of the benzimidazole ring affected tau protein affinity, which has enabled development of new radiotracers with higher affinities, such as [18F]N-methyl lansoprazole [53]. This research field is based on the study of lansoprazole, and indeed its high affinity to tau protein is a striking and open avenue for researchers to create and improve noninvasive techniques for diagnosing AD in the early stages. Many of these studies are still in preclinical or early clinical trial stages. Further investigations are needed to specifically understand PPI interactions with tau protein.

### 3.3. PPIs and Vitamin B_12_ Deficiency

Gastric acidity is necessary for absorption of vitamin B_12_, which is an essential water-soluble vitamin obtained from different dietary sources such as fish, meat, dairy products, and fortified cereal [56]. Statistically, approximately 6–20% of American adults have vitamin B_12_ deficiency, likely as a natural phenomenon. Nonetheless, the risk of B_12_ deficiency increases with age [57]. During digestion, vitamin B_12_ binds to salivary R proteins and then to intrinsic factors, reaching the whole intestine and ileum terminal intact, where B_12_ absorption occurs. B_12_ is firmly bound to proteins and consequently requires acid-activated proteolytic digestion. Use of PPIs causes hypochlorhydria, which results in vitamin B_12_ malabsorption as B_12_ remains tightly bound to proteins in the stomach [58]. Several studies have shown controversial results between long-term PPI use and vitamin B_12_ deficiency. For instance, in a case-control study, patients treated with PPIs for 2 years or longer showed a statistically significant association with increased risk of B_12_ deficiency [59]. In contrast, in a cross-sectional study, patients prescribed PPIs for 3 years or longer had similar B_12_ levels as non-PPI users [60]. It is important to note that most of these trials show association and not causation. Likely, other factors will contribute to these findings in addition to acid-suppressive therapy [61].

Vitamin B_12_ deficiency caused by PPI use has been associated with dementia and cognitive impairment [62]. Vitamin B_12_ is required for one-carbon transfer reactions such as methylation, which are needed for processing and production of nucleotides, phospholipids, and monoamine neurotransmitters [63]. Usually, vitamin B_12_ removes a methyl group from tetrahydrofolate, turning it into methylcobalamin. Methylcobalamin then presents its methyl group to homocysteine, which is finally converted to methionine by methionine synthase [62]. Thus, vitamin B_12_ deficiency is one of the main causes of hyperhomocysteinemia. Both hyperhomocysteinemia and B_12_ deficiency are considered risk factors for brain atrophy, cognitive impairment, and dementia [64]. Studies have shown that hyperhomocysteinemia may activate several protein kinases, such as glycogen synthase kinase 3*β* (GSK-3*β*), cyclin-dependent kinase-5 (Cdk-5), c-Jun N-terminal kinase (JNK), extracellular signal-regulated kinase (ERK), and p38 mitogen-activated protein kinase (MAPK), and inhibit protein phosphatase 2A (PP2A) (41), which are all pivotal enzymes in regulating the phosphorylation state of tau protein [65]. Among these enzymes, PP2A plays a crucial role as it is the main brain serine/threonine phosphatase and prevents tau hyperphosphorylation [66]. Decreased methylation may affect PP2A function by leading to hyperphosphorylation and aggregation of tau protein [62]. In animal models, aside from inducing tau protein hyperphosphorylation, hyperhomocysteinemia can increase A*β* production, while folate/vitamin B_12_ supplementation may attenuate these effects [67–69]. Based on these findings, high homocysteine levels are a strong and independent risk factor for developing AD [70]. Alternative mechanisms for linking AD with vitamin B_12_ deficiency have also been described, which are distinct from PP2A inactivation and tau hyperphosphorylation. Using the structure and function of vitamin B_12_ inside cells, Rafiee and colleagues (38) [62] studied direct binding between B_12_ and tau protein *in vitro* by fluorometry and circular dichroism. Because vitamin B_12_ can interact with thiol groups, they determined that cobalamin can directly bind to tau via cysteine residues on tau protein. Hence, the resulting B_12_/tau protein complex prevents tau protein fibrillation. Besides, tau aggregation is inhibited by vitamin B_12_ capping on cysteine residues of tau. In summary, the effect exerted on neurodegeneration by vitamin B_12_ deficiency and hyperhomocysteinemia is not only due to PP2A inactivation and tau hyperphosphorylation but also direct binding of vitamin B_12_ to tau protein, inhibiting its fibrillation and aggregation [62].

Although different mechanisms have been described to explain the effects of vitamin B_12_ deficiency in dementia, more clinical trials are needed to understand this relationship. In addition, more studies are required to establish if vitamin B_12_ deficiency is a causal event in dementia or an associated factor, as shown in other studies.

## 4. PPIs and Antineurotoxicity

In contrast to the effect of PPIs on A*β* plaque production, tau protein, and vitamin B_12_ deficiency in AD development, different studies have shown antineurotoxic effects of PPIs on astrocytes and microglia [17]. In AD patients, activated astrocytes exist in close relationship with senile plaques and neuronal degeneration [71]. These activated cells release powerful neurotoxic products, including proinflammatory cytokines, reactive oxygen species, and nitric oxide [72, 73]. Although astrocytes show neuroprotective activity, activated astrocytes may aggravate neurodegenerative diseases [74]. Hashioka et al. [17] demonstrated that lansoprazole and omeprazole decrease interferon- (IFN-) *γ*-induced astrocytic neurotoxicity as a result of inhibition of signal transducer and activator of transcription 3 (STAT3) activation. They also confirmed that PPIs slightly reduce production of T-cell alpha chemoattractant (I-TAC) by IFN-*γ*-activated astrocytes, although only lansoprazole shows a significant effect. These findings are in agreement with another study by Hashioka et al. [18] showing that lansoprazole and omeprazole may suppress tumor necrosis factor- (TNF-) *α* production from THP-1 cells and decrease human microglial and monocyte neurotoxicity. In addition, they showed that nonsteroidal anti-inflammatory drugs (NSAID) can increase antineurotoxic effect of PPIs due to their synergic effect. Furthermore, a recent study identified lansoprazole as a liver X receptor (LXR) agonist [75]. LXRs are transcription factors that modify expression of genes related to cholesterol metabolism. Hence, lansoprazole increases ATP-binding cassette transporter (Abca1) and apolipoprotein- (Apo-) E levels in primary astrocytes, both genes regulated by LXR [75]. Moreover, ApoE lipid complexes mediated by ABCA1 inhibit A*β* plaque aggregation, thereby supporting the theory that lansoprazole can act as a therapeutic agent in AD [17].

## 5. Clinical Association between PPIs and Risk of Dementia, AD, and Cognitive Impairment

Based on a systematic review from 2017, four European observational studies have investigated association between PPI use and dementia. Three studies have found a positive association between dementia and omeprazole, esomeprazole, lansoprazole, and pantropazole, with an approximately 1.4-fold increased risk of any dementia in cohorts using PPIs (95% CI, 1.36–1.52; *P* < 0.001) [76]. Similarly, in a recent prospective cohort study in Asian population (*n* = 15726, 7863 PPI users), an association with dementia has been found (aHR, 1.22; 95% confidence interval, 1.05–1.42) [15]. In contrast, the fourth European report included in the systematic review found a negative association (OR dementia with PPI use = 0.94 (95% CI, 0.90–0.97) *P* = 0.0008) [76]. Herghelegiu and colleagues [77] conducted a single-center case-control study that compared 148 PPI users (aged at least 65 years old) with a control group of nonusers during an 8-month period. They confirmed statistically significant association between PPI use and dementia. However, bias was influenced by the small study sample. Further, they did not take into account fundamental cofounders such as age, sex, history of stroke, and smoking status, which are considered risk factors for dementia. Conversely, Booker and coworkers [16] found statistically significant reduction between dementia and PPI use with a case-control study using a database of general practice medical records in Germany and including 11,956 patients with initial dementia diagnoses over a 4-year period. This study was considered to have a moderate risk of bias due to codes being entered by general practitioners. The other two cohort studies performed by Haenisch et al. and Gomm et al. [9, 14] used data from two different German databases (KNDD and AgeCoDe, resp.) which reported positive association between PPI use and dementia. Although both studies included a wide range of cofounders, neither took into account either hypertension or family history of dementia (again well-known risk factors for dementia) nor sugar intake, physical exercise, or air pollution, recently considered risk factors for dementia [76]. In contrast, Haenisch et al. [14] performed the only study to include a subgroup for evaluating AD risk. Their results were in favor of elevated risk for AD associated with PPI use. Besides, exclusion of cofounders (such as age, sex, education, ApoE4 allele status, polypharmacy, depression, ischemic heart disease, and stroke) did not influence the effect of PPI use on AD.

In the same systematic review, weak association between PPI use and acute cognitive impairment was demonstrated in a series of case reports and small observational studies [76]. Regarding these, four were hypomagnesemia-associated delirium or confusion cases [78, 79], one was associated with hyponatremic delirium [80], and one was delirium of unknown cause [35]. PPIs were implied as a main cause in these effects because they can lower magnesium and sodium levels, and the majority of studies were associated with omeprazole use [35, 78, 80]. In one case, withdrawing esomeprazole therapy provided symptomatic relief and reestablishment of magnesium levels [78]. With these observational studies, data on PPI use and risk of delirium was incorporated [81, 82]. For instance, Otremba and coworkers performed a cross-sectional study, including 675 patients of 60–100 years old, who were admitted to a subacute geriatric ward over a 12-month period. They found that PPI use was a predictive factor for developing delirium in these patients. The Confusion Assessment Method and Delirium-O-Meter were the scales used for diagnosing delirium and its severity, respectively [82]. However, Fujii and coworkers, who compared incidence and severity of delirium in H(2) blocker users and PPI users, found that delirium can be reduced by switching H(2) blockers to PPIs [81]. However, there are major difficulties in evaluating these studies as the data does not contain reliable information. Also, the majority of studies used different group characteristics, which makes comparisons among them challenging [76].

Other studies have focused on short-term PPI use and its influence on different cognitive functions. For instance, Akter et al. used the Cambridge Neuropsychological Test Automated Battery (the well-known CANTAB software) to evaluate each PPI and its effect in different cognitive domain functions of young patients (20–26 years old) over 7 days [12]. CANTAB software can accurately determine amyloid-related cognitive decline and quantify the severity of impairment in prodromal AD patients [83]. This study found that taking different PPIs may influence several degrees of cognitive capacity. For instance, omeprazole led to deterioration of visual and episodic memory, motor and mental response speed, new learning, short-term and sustained attention, retention and manipulation of visuospatial information, and strategy development [12]. Lansoprazole, in addition to the mentioned effects of omeprazole, can restrict manipulation of remembered memory to generate a complex task or strategy and also limit retention of spatial information. Contrary to lansoprazole and omeprazole, esomeprazole induced difficulties in maintaining sustained attention, retaining and manipulating spatial memory, and planning strategy [12]. In contrast, a very recent study including data from the Nurses' Health Study II has not shown association between PPI use and cognitive dysfunction or dementia risk. However, this study indicates a modest association for psychomotor speed and attention among PPI users [84]. To explain some acute and chronic cognitive effects, it is possible that PPIs may preferentially affect the hippocampus and associative neocortex via a neuroplasticity mechanism. Still to our knowledge, there is no evidence of *in vivo* or *in vitro* experiments using long-term potentiation protocols to confirm this theory.

## 6. Final Considerations and Conclusion

There is currently no consensus on the role of PPIs and the associated risk of developing dementia. Because of the multifactorial origin of dementia (Alzheimer–vascular spectrum dementia), future studies are required to consider associated environmental and genetic factors, as well as biomarkers (i.e., APOE-*ε*4) and other covariates (i.e., chronic stress) that may increase the risk of dementia in patients who consume PPIs. It is possible that the cognitive effects of PPIs are due to drug interactions, especially in polymedicated elderly patients. For instance, omeprazole may increase blood levels of diazepam (a *γ*-aminobutyric acid [GABA]-A agonist) by decreasing plasma clearance (via cytochrome P450) and then increasing neurological side effects [85]. In a retrospective study in six residential care homes in England (*n* = 133), it was found that 9.2% of older people with dementia were prescribed with two or more potentially inappropriate medications, including PPIs and long-acting benzodiazepines [86]. In addition, an FDA study has shown that some adverse events with PPIs (including omeprazole, lansoprazole, and pantoprazole) could be associated with benzodiazepine drug interactions [87]. As long-term benzodiazepine use might increase dementia risk [88, 89], it is possible that combined treatment of GABA-A agonists and PPIs may increase this susceptibility, considering the close association between GABAergic system dysfunction and the physiopathology of AD and mild cognitive impairment [90]. In fact, cognitive impairment has been reported after lorazepam treatment in patients with higher risk for AD (APOE-*ε*4 allele carriers) [91]. At recommended doses, a pharmacokinetic interaction between benzodiazepines and PPIs is less probable with pantoprazole, lansoprazole, and rabeprazole than with omeprazole [92–94]. Accordingly, those PPI drugs might be considered in polymedicated subjects.

Cumulative evidence indicates that chronic treatment with NSAIDs (e.g., ibuprofen) may delay AD onset and reduce AD rate of progression [95, 96]. As mentioned earlier, *in vitro* evidence indicates that PPIs might have neuroprotective and anti-inflammatory effects that act synergistically with NSAIDs. Some limitations of *in vitro* studies include the experimental duration, as well as complex specific microenvironment factors observed only *in vivo* [97]. In fact, dementia and AD are chronic neurodegenerative diseases with a complex physiopathology and several compensatory neuronal-glial mechanisms after long-term A*β* peptide exposure. To simulate environmental cellular conditions of AD, it is necessary to design durable *in vitro* studies that involve persistent oxidative stress and metabolic dysfunction.

Vascular and BBB dysfunctions have been observed in AD patients [98]. Taking into account age-related changes of BBB function, as well as vulnerability to disruption by external factors such as hypertension and drugs [99, 100], we suggest that neurological susceptibility to PPIs may be related to changes in BBB permeability, as well as changes in the brain microenvironment related to aging. However, more studies are necessary to identify other factors that contribute to this susceptibility.

Although the mechanisms of brain dysfunction induced by PPIs are not known with certainty, it is possible they influence ionic pumps controlling the membrane potential and electrochemical gradient in neurons. Considering the preferential effects of PPIs on A*β* and tau protein, as well as on endothelial function, further studies are needed to contemplate differential susceptibility between AD and vascular dementia. Taking into account the effects of PPIs on vitamin B_12_ levels, and possibly indirect effects on membrane ionic transporters, nutritional and electrolyte monitoring is required in patients who chronically use PPIs, mainly older adults and patients with chronic malnutrition or debilitating chronic conditions. Also, it is necessary to determine the previous cognitive status of patients and whether they have risk factors for dementia, as well as pharmacokinetic drug interactions. Altogether, it is necessary to consider the risk–benefit of chronic PPI use and, above all, strictly establish an adequate therapeutic indication.
